# Supplementary material for: Knowledge, attitudes, practices of/towards COVID 19 preventive measures and symptoms: A cross-sectional study during the exponential rise of the outbreak in Cameroon
Source: PLoS Negl Trop Dis. 2020 Sep 4;14(9):e0008700. doi: 10.1371/journal.pntd.0008700 (PMC7497983; doi:10.1371/journal.pntd.0008700)
Supplement: S1 Appendix — (DOCX) [file pntd.0008700.s001.docx]

**S1 Appendix: Survey questionnaire**

**KNOWLEDGE, ATTITUDES, PRACTICES ON COVID 19 PREVENTIVE MEASURES AND SYMPTOMS.**

Dear participant, we are researchers at the Center of Medical Research, Cameroon. This voluntary KAP and symptomatology questionnaire was conceived in order to help the government in the fight against the dreaded Corona virus pandemic. We wish that you accompany us in this goal by providing us with accurate answers for these questions. All responses will be kept anonymous and confidential.

This will take just 10 mins of your time and kindly forward it to your contacts. In case you have any inquiries please contact [ngondechan@yahoo.fr](mailto:ngondechan@yahoo.fr) and ngwaadela@gmail.com. Thank you for your cooperation.

Cher participant, nous sommes chercheurs au Centre de Recherche Médicale du Cameroun. Ce questionnaire KAP/symptomatologie est volontaire et a était conçue afin d'aider le gouvernement dans la lutte contre la redoutable pandémie du virus Corona. Nous souhaitons que vous nous accompagniez dans cet objectif en nous fournissant des réponses précises à ces questions. Toutes les réponses resteront anonymes et confidentielles.

Cela ne prendra que 10 minutes de votre temps et nous vous prions de faire suivre a vos contacts.

Pour plus d'informations, veuillez contacter ngondechan@yahoo.fr et ngwaadela@gmail.com. Merci de votre collaboration.

**SECTION A-Demographics (Démographie)**

1) City of residence (Ville de résidence): ……………………………………..

2) Gender (Sexe) : Female (Féminin) Male (Masculin)

3) Age……………………………………………………………………………

4) Profession…………………………………………………………………….

5) Which of the following best describes your working environment? Lequel des énoncés suivants décrit le mieux votre environnement de travail?

- Hospital milieu (Milieu hospitalier)
- Office confinement (Confinement de bureau)
- Face to face interaction with customers (Interaction face à face avec les clients)
- Out door environment (Environnement extérieur)
- Home environment (Environnement de la maison)

**SECTION B-Source/ period of information on COVID 19**

6) When did you first hear of COVID 19? Quand avez-vous entendu parler de COVID 19 pour la première fois?

- Decembre 2019 (Decembre 2019)
- January 2020 (Janvier 2020)
- February 2020 (Février 2020)
- March 2020 (Mars 2020)
- Never heard (Jamais entendu parler)
- Others (Autre) :………………………………………………………………………

7) How did you first get the information on COVID 19? (Comment avez-vous obtenu les informations sur le COVID 19 pour la première fois)

- Newspaper (Journal)
- Websites (Sites web)
- Whatsapp
- Television (La télévision)
- Mouth to mouth (bouche à oreille)

**SECTION C-Knowledge on disease transmission (Connaissances sur la transmission de la maladie)**

**No(Non)**

**Yes (Oui)**

8) By droplets when infected person coughs, sneezes or speaks

(Par gouttelettes lorsqu’une personne infectée tousse/éternue)

9) By kissing infected person

(Par Bisou ou embrassade d’une personne infectée)

10) By touching a contaminated surface and then touching your face

(En touchant des surfaces infectées ensuite vôtre face)

11) By handshake (Par poignéee de main)

12) By blood transfusion (Par transfusion sanguine)

13) By sexual intercourse (Par rapport sexuels)

14) By Contaminated foodstuffs (Par danrée d’aliments contaminée)

**SECTION D- Attitudes towards COVID 19 health seeking behavior**

15) If you are living with someone working in a hospital milieu do you think they can contaminate you? Si vous vivez avec un proche qui travaille a l'hôpital penser vous qu'il peut vous contaminer

Yes (Oui) No (Non)

16) Are you willing to do a voluntary test for COVID 19? Êtes-vous disposé à faire un test volontaire du COVID 19?

Yes (Oui) No (Non)

17) If you have another disease other than COVID 19, will you go to the hospital? Si vous avez une autre maladie que le COVID 19, vous rendez vous a l'hôpital pour une consultation?

Yes (Oui) No (Non)

If No, why? Si non, pourquoi?..............................................................................................

18) Do you prefer to be confined in the house or hospital for your medical care when you are tested positive for COVID 19? Préférez-vous être confiné à la maison ou à l'hôpital pour vos soins médicaux lorsque vous êtes testé positif pour COVID-19?

- House (Maison)
- Hospital (Hôpital)
- I don't know (je sais pas)

Please explain the choice of staying at home (Veuillez expliquer la raison de votre choix de rester à la maison) ………………………………………………………………………….

**SECTION E: Practices on preventive measures (Pratiques sur les measures preventives)**

What are the measures you are currently taking to prevent this illness? (Quelles sont les mesures préventives que vous prenez actuellement pour éviter cette maladie?)

**No(Non)**

**Yes (Oui)**

19) Social distancing (La distanciation sociale)

20) Washing hands and using sanitizers

(le lavage des mains et l’utilisation des gels hydroalcooliques)

21) Total confinement (Le confinement total)

22) Use of mask (l’utilisation des masques)

23) Use of traditional concoctions

(l’utilisation décoctions traditionnelles)

24) Taking chloroquine (Prendre de la chloroquine)

25) Eating citrus fruits such as lemon and taking Vitamin C

(Manger des agrumes comme le citron et Prendre de la vitamine C)

26) Taking paracetamol (Prendre le paracétamol)

27) Taking Ibuprofen (Prendre l’Ibuprofène)

**SECTION F: Symptomatology (Symptomatologie)**

28**)** Which of the following symptoms do you have? (Lequels de ces symptômes présentez-vous ? **(**Multiple options/Choix multiples)

- Fever (Fièvre)
- Dry Cough/ catarrh (Toux sèche/ rhume)
- Throat irritation (Irritation de la gorge)
- Headache (Maux de tête)
- Diarrhea (La diarrhée)
- Muscle pain (les douleurs musculaires)
- Do not smell odor or taste (Perte de goût/ d’odorat)
- Difficulty breathing (Difficultés respiratoires)
- None of the above (Aucune de ces réponses)

29) Do you usually have the above symptoms frequently? (Présentez vous ces symptômes de manière fréquente).

Yes (Oui) No (Non)

30) When you have these symptoms, do you call 1510? Lorsque vous présentez ces symptômes, appelez-vous le 1510?

Yes (Oui) No (Non)

31) Which of the following diseases are you currently suffering from? (Souffrez-vous de l'une des maladies suivantes?) **(**Multiple options/Choix multiples)

- High blood pressure (Hypertension artérielle)
- Cancer (Le cancer)
- Diabetes (Le diabète)
- Cardiovascular diseases (Maladies cardiovasculaires)
- Asthma (Asthme)
- Renal failure (Insuffisance rénale)
- None of the above (Aucune de ces réponses)

32) Have you been recently diagnosed of any of the following diseases? (Avez-vous été diagnostiqué récemment d'une des affections suivantes) (Multiple options/Choix multiples)

- Respiratory tract infection (Infection respiratoire)
- Common flu (grippe commune)
- Allergic cough (Toux allergique)
- Tuberculosis (Tuberculose)
- Malaria (Paludisme)
- None of the above (Aucune)
