# Supplementary material for: Knowledge, attitudes, practices of/towards COVID 19 preventive measures and symptoms: A cross-sectional study during the exponential rise of the outbreak in Cameroon
Source: PLoS Negl Trop Dis. 2020 Sep 4;14(9):e0008700. doi: 10.1371/journal.pntd.0008700 (PMC7497983; doi:10.1371/journal.pntd.0008700)
Supplement: S1 Table — (DOCX) [file pntd.0008700.s002.docx]

**S1 Table: City of residence of respondents**

| Regions | Cities | Number of respondents |
| --- | --- | --- |
| Adamawa | Ngaoundéré | 7 |
| Northwest | Bambili, Bambui, Bamenda, Kumbo, Ndop | 41 |
| West | Bafang, Bafoussam, Baham, Dschang | 21 |
| East | Bertoua | 5 |
| Southwest | Buea, Kumba, Limbe, mutenguene, Tiko | 111 |
| Extreme North | Maroua | 6 |
| Littoral | Douala, Nkongsamba | 152 |
| North | Garoua, Garoua boulai, Doume | 10 |
| Centre | Yaounde, Monatélé, Soa, Okola | 640 |
| South | Kribi, Sangmelima, Ebolowa | 13 |
