# Supplementary material for: Knowledge, attitudes, practices of/towards COVID 19 preventive measures and symptoms: A cross-sectional study during the exponential rise of the outbreak in Cameroon
Source: PLoS Negl Trop Dis. 2020 Sep 4;14(9):e0008700. doi: 10.1371/journal.pntd.0008700 (PMC7497983; doi:10.1371/journal.pntd.0008700)
Supplement: Attachment — Submitted filename: Responses to reviewers comments Plos COVID (1)E.docx [file pntd.0008700.s003.docx]

**RESPONSES TO REVIEWERS COMMENTS (Manuscript No: PNTD-D-20-01057)**

Reviewer #1: The objective as well as the hypothesis are both clearly stated. The challenge for the authors is to more clearly and strongly link the results to the hypothesis that the KAP can indeed impact the acceptance of recommended measures to interrupt transmission of the corona virus. One area that is not clear to me is the collection of information on symptomatology. This avenue of study did not clearly fit into the stated objectives as there is not effort made to tie individuals’ symptoms into the behaviors that a KAP seeks to establish. My recommendation is to look at whether self -reported symptoms plays a role in any of the three pillars of a KAP.

**Response**

Thank you for the question. It has been stated in the abstract line 17-19 and Table 8 has been included. We agree that the avenue of symptomatology is usually not an aspect of KAP studies. However, this new aspect of symptomatology we are putting forward is intricately linked to KAP in the following ways:

1. Do they know about the disease (information on symptoms, origin gotten from television and other sources) and how it is transmitted (Knowledge)
2. Do they seek for help/adhere to norms when they present with symptoms of COVID 19 (Attitudes)
3. What do they practice to treat these symptoms or prevent the illness (Practice) like taking auto-medications, wearing masks, taking traditional concoctions, social distancing etc

The questionnaire is found in the appendix section

The study design is appropriate given that it was conducted during a time of partial confinement and self-quarantining.  There are further comments on this in the discussion of the study’s limitations.  The population is clearly described though (and again to be discussed later in the limitations section) the population is limited to urban dwellers possessing the necessary technology and are literate.  This corresponds with the fact that apparently all respondents were employed with perhaps the exception of those listed under “other”. These factors are ones that may exclude those who are more vulnerable to disease i.e. those who are not as socially, economically advantaged. These factors should be noted in the study population and eligibility criteria section as well as in the discussion of limitations.

**Response**

Thank you. The population is now clearly described in the study population and the eligibility criteria section (Line 148) and ‘others’ on table 1 have been clearly defined as housewives, builders, taxi drivers, unemployed, farmers.

It is worth noting here that this disease was imported with the first cases being reported in cities and was not a common phenomenon in Cameroon. Also, the cities are denser than the villages and people interact more in cities with the use of taxis, open business places, etc. Taking the case of the Northwest region of Cameroon, we notice that COVID 19 is more prevalent in the cities than in the villages like in Bamenda city as compared to Ako and Ndu villages.

I would also recommend that the authors provide more context of the disease within Cameroon citing available statistics and explicitly stating what measures the government has taken to slow transmission. This would be much more helpful than the global overview of the pandemic that is given.

**Response**

Thank you this has been cited in the manuscript. Line 76-85

I defer the questions concerning sample size and statistical analysis to those who with more expertise than my own.

There are no ethical concerns though the authors could state if any recommendations and actions were made to those reporting symptoms that could indicate infection with the corona virus.

**Response**

Thank you. This online study was anonymous and confidential. It was a survey to detect the level of suspected cases and alert the ministry of public health on the urgency to carry out rapid interventions in the areas surveyed. So a report was made through my institute to the Ministry of public health for further actions to be taken.

Reviewer #2: • The objectives stated are very superficial and no hypothesis are stated in the manuscript.

**Response**

Thank you. The objectives and hypothesis are stated in the manuscript, Line 17-19, 118-121

Hypothesis:

a) Demographic variables of Cameroonian residents could influence KAP and symptomatology, and

b) KAP could influence the risk of having COVID19.

It appears one of the attempts researchers made is to test the association of demographic characteristics on KAP.  This part needs major revision of stating all the hypothesis testable from current data and articulating objectives.

**Response**

Thank you this has been adjusted Table 4 and 5

•    Study design to be improved stating dependent vs  independent variables being studied  with reference to  each of the objective

**Response**

The objectives of the study were to:

1. Evaluate the factors influencing the knowledge, attitudes, and practices on the spread of COVID 19
2. Evaluate the associations between the demographics, KAP and symptoms of COVID 19 (Line 118-121)

.

Variables are:

KAP and symptomatology are dependent and demographics is an independent variable

KAP is the dependent variable, symptomatology is the independent variable

•    It seems study sample is taken from 4  cities, of  above 18 years age,  with WhatsApp, email & website facility.  Details of total population of the 4 cities viz. population size, salient demographic and social features, economic and educational status and incidence/prevalence of covid-19 etc be given.

Unless there is adequate justification to say this sample represents the general population of the 4 cities, the findings will be applicable to only to the sample category of the population.  This may be one of the limitations. For the current visible hypothesis sample size would be adequate.

**Response**

The current economic status, social features, etc of each city could not be obtained given that it has not been updated for over 15 years. The prevalence of COVID19 per city could not be obtained. We could only have per region.

|  | Yaounde (centre) | Douala (Littoral) | Buea (southwest) | others |
| --- | --- | --- | --- | --- |
| Greater than 3 symptoms | 28 | 7 | 3 | 3 |
| Prevalence of COVID 19 on 5th of May per region | 1280 | 771 | 33 | 721 |

•    The conclusions need to be rewritten based on the analysis which has to be in accordance with the  objectives

**Response**

Thank you this has been done

To be mentioned briefly in the methodology:

•    Category of respondents, proportion of the sample (affected/healthy/suspected), Sampling procedure  applied.

**Response**

Thank you we were looking at the suspected cases only given that no standard tests were done to confirm healthy and affected cases.

•    The activities mentioned in the manuscript  like- suspecting the cases of covid-19, Assessing their  symptoms, collecting the details of comorbidity etc  are carried out as part this research study ?  Whether primary or secondary methods used for every data collected.

**Response**

Thank you. We were interested in reported symptoms by patients only. Also, people who reported with comorbidities like hypertension, diabetes know their current status since these are chronic illnesses.

•    Clarify possible range of min-max KAP scores. If the scores (mentioned in line 144) 3-7, 1-4, 2-9 are achieved ones it is possible  there are some false questions, which may be deleted from manuscript as they will be misleading the readers.

**Response**

Thank you the low scores of 0-3, 0-2 were attributed to the false answers. It is in the questionnaire it is represented as F in the legend. S1 Appendix

Details of  analysis  done mentioning the types tables  and  statistical tests used for testing  each of the two variables.

**Response**

Thank you this is found under Study design (Line 135-140 and 180-187)

Reviewer #3: The paper employs an online survey with offline recruiting to study the Knowledge, Attitudes, and Practices related to the COVID-19 in Cameroon. The analysis is ok, and the interpretation of the results is proper. My only concern with methods are the following:

1. The recruiting is problematic: the respondents were recruited by a poster that invited people to answer the survey. This builds in a bias inside the responses, dismissed because the authors find the disease's prevalence similar to the reported number of cases. I believe this is still problematic, and the authors have to point out some problems and some strengths of this 'snowballing sampling' process.

Suggestion 1: Explain the recruiting process better and have an appendix with an example of the poster used for recruitment. Moreover, the word poster somehow gave me the idea that it was something physically placed somewhere. Luckily I read the paper twice, but some other people may have the same perception.

Suggestion 2: There certainly are strengths in snowballing a sample. The authors acknowledge that the sampling may be a limitation, but they could also discuss how it may be advantageous: cheap and feasible in a pandemics situation. This has to be stressed and perhaps suggested for similar studies.

**Response**

Thank you. the recruitment sheet has been included in the appendix. This method was also very cost effective and feasible given that the data was collected during a period of confinement.

Thank you this has been included in the discussion section Line 310-312.

2. The demographics: the authors should provide the demographics of the country and how they relate to their sample demographics. As their sample comes from people that know how to read, perhaps they should take the country demographics with these proper restrictions.

Suggestion: find out the country demographics and discuss how they relate with the survey one. One caveat here is that the last Census was taken in 2005. However, the authors could find projections in the BUCREP website (or at least explain why not possible to find reliable aggregated country-level data).

**Response**

Thank you very much for this insightful point. There is no available data for the current census of Cameroon and the last census was carried out in 2005 when the population was about 15 million inhabitants. Now with an estimated population of about 25 million we couldn’t do the post stratification because it will include bias in our analysis.Line 417-419

3. Answering time analysis: As the COVID grew over time, so did the media exposure and the knowledge from people. They had about one month of data collection going on. Did the patterns change from the first to the last 15 days? If so, how?

Suggestion: redo the analysis using the split sample of SPSS by dates, breaking in two or three subsequent periods.

**Response**

Thank you for this insightful suggestion. It has been done (Table 2). The main source of information was through the television, followed by social media then mouth to mouth and it did not change over time.

4. Multilevel Regression and Post-stratification: Based on suggestions 2 and 3, you could also run a multilevel regression and poststratification on the covariates in a census to see if you could reweight the sample, to make the sample closer to the actual population in Cameroon.

Suggestion: check to see if you could apply multilevel regression, and have better estimates by each town or region. If not, at least raking the sample to make it closer to the proportions in the population. This piece might help to do the raking using SPSS ([https://community.ibm.com/HigherLogic/System/DownloadDocumentFile.ashx?DocumentFileKey=17fd2f0b-7555-6ccd-c00c-5388b082161b&forceDialog=0](about:blank)).

**Response**

The national census for Cameroon has not been carried out since 2005 and the projections from this year may not truly represent those of 2020. Now with an estimated population of about 25 million we couldn’t do the post stratification because it will include bias in our analysis.Line 417-419

Should these points be addressed, I believe the paper will increase considerably in quality and breadth.

Minor points:

1. What program did you use to collect the answers? If Qualtrics, you have georeferenced information from the respondent.

**Response**

We used Google forms. The use of qualtrics has been mentioned as a recommendation in the conclusion section (line 391) for future studies to help locate respondents with symptoms and better manage cases in particular circumscriptions.

2. Have you incentivized the responses in any way?

**Response**

No

3. I am not an expert on Cameroon, but is there any social media that could have been used to boost the response rates?

**Response**

Cameroon does not have a local social media platform. We make use of WhatsApp only.

Reviewer #1: The analysis plan is followed as far as it goes. The reader is presented with the findings of concerning knowledge, attitude and practices through tables that are sufficiently clear. I would recommend avoiding the terms of good and right and wrong knowledge and other somewhat judgmental adjectives.  The fact that the global understanding of the virus is in flux, what is seen as “good” may not be in another month.  The authors should present to us what their baseline is for judging behaviors and knowledge. An example is that whether hospital seeking behavior should be considered as a positive behavior considering that nosocomial transmission is indeed a risk.

As noted above, the symptomatology does not provide any additional insight into the results of the KAP.  I would recommend that if possible, the authors analyze the symptomatology data in light of the other findings of the actual KAP.  Does having symptoms impact on attitude, on practice, etc.?

**Response**:

Thank you. The judgemental adjectives have been removed in the manuscript.

We do agree that the avenue of symptomatology is usually not an aspect in KAP studies. This new aspect of symptomatology we are putting forward is intricately linked to KAP in the following ways:

1. Do they know about the disease (information on symptoms, origin gotten from television and other sources) and how it is transmitted (Knowledge)
2. Do they seek for help/adhere to norms when they present with symptoms of COVID 19 (Attitudes)
3. What do they practice to treat these symptoms or prevent the illness (Practice) like taking auto-medications, wearing masks, taking traditional concoctions, social distancing etc

The questionnaire is found in the appendix section

Reviewer #2: Analysis needs major modifications, and also will have to be articulated as  per the  modified objectives and analysis plan:

Table-2  can bring out a crucial finding  if it can be made a cross table  (source vs period)  to asses the magnitude of effective and early means of communications among the study population.

**Response:**

Thank you. This has been done.

Table-3: shows the response of total sample to the  KAP questionnaire.  No need to show the question as such  in the table. Need to provide N value  wherever only % is given.

**Response:**

Thank you. I think the questions represent the factor or variable we are assessing so it can be maintained in the table.

•    Table-4  seems to be  primarily used for testing the association between level of knowledge of the total sample and demographic variables, which needs percentage corrections.  Cross tabulations with independent variables in rows need to show  row percentages, so that as in case of gender (table-4)  it can be inferred, the proportion of respondents with high  or low knowledge is more or less from  which gender.  Column percentages presently showing only the gender proportion, not of those with high and low knowledge in each gender. In the same row  the  test used for significance and (p=  ) value details etc be mentioned.  Mean scores has no importance.

**Response**

Thank you, table 4 has been corrected

•    Similar tables be developed for testing any associations if in the objectives between any variables and the levels  attitudes, practices, Symptomatology, Comorbidity  with title specifying  the association being tested  in the table.

**Response:**

Table 8 has been added to the manuscript (Associations between symptomatology and KAP). The comorbidity assessment is secondary to the symptoms of COVID 19 since we were interested in COVID primarily and the number of those suspected for COVID 19 with underlying comorbidities.

•    Avoid using of  value judgments  by  using ‘low knowledge(0-3)’    and  ‘high knowledge(4-7)’  or  adequate knowledge (        )  and inadequate  knowledge(      ) mentioning the score ranges instead of  ‘right knowledge’  and ‘wrong knowledge’  in table-4.  Similarly  avoid  using  terms like  ‘good practice’  & ‘wrong practice’  in table-5; positive and negative  practices  or risky or non risky /traditional  and modern practices      which  are used in discussion may be used.

•    All the tables like table-7, should  show totals or  (N= )  in proper place .  From page-15 under symptomology  need to specify the symptoms of  what disease.

Findings from each of the figures and tables be clarified  as per objectives.

**Response**

Thank you this has been corrected in the whole manuscript

Reviewer #3: The results are clear and intelligible. The tables and images are of excellent quality. The interpretations are precise and match the results in the tables. The writing is clear and concise, and I thank the authors for having written such a pleasant paper to read.

**Response**

Thank you very much for the complement

--------------------

Reviewer #1: I find the discussion and conclusion sections to be in need of greater thought particularly in regard to the public health implications.  I congratulate the authors for undertaking this work rooted in behavioral health as these are the elements that prior to the development of therapeutic drugs and vaccines that will reduce the infection and mortality rate. The data provided offers a number of avenues to explore within a public health context which need much more critical analysis. For example, In the last line of the first paragraph in the discussion section, it was stated that …”potentially risky behaviors were related to the female gender’’’”.  This finding (which also needs to be better justified) opens up a number of doors for interventions with women as the target audiences whether it is communication initiatives, educational approaches, etc.  The authors suggest a confidential on-line system to share experiences. In making this recommendation, the data

from the KAP should be used by the authors to justify the idea.

My overall recommendation is for the authors to tie the data more directly into interventions that could be recommended to governmental and non-governmental organizations.

**Response**

Thank you this has been revised in the conclusion section

In terms of limitations, I think the authors overlook a number of issues here.  Primarily, the use of the technology limits participation to those who have access to the various platforms used.  Another limitation is the study depends on respondents being literate which also excludes many of Cameroon’s poor and as we see in various countries disparities along socio-economic lines of who gets infected are very clear.  The relatively high % of people taking recommended precautionary measures could easily be related to their educational level, income, etc.

Though the disease may initially be more prevalent in cities, we have seen in other settings the migration to home villages bringing infection with them.  Their recognition of the need for further work at the grass roots level is well taken and I would recommend it being a recommendation in the conclusion or discussion section.

**Response**

This has been included in the discussion and conclusion section. Also the others described in table 1 includes housewives, farmers, unemployed less priviledged people

Additionally, I would suggest that if the authors are not prepared to recommend interventions based on the results, they state that a limitation to the study is that a KAP is a quantitative tool and that to really focus on behavior change qualitative work would be necessary.

**Response**

Thank you this has been stated in the limitation section Line 410-411

The final limitation of the study is the constant flux in the global knowledge of Covid-19 and what is considered best practices.

**Response**

Thank you this has been corrected in the whole manuscript

Reviewer #2: The conclusion- People’s attitudes and practices could stem from panic and stigma- to be verified whether it is out of stigma or  provided knowledge to them. Rest of the conclusions are suggestions to the programme, addressing them with online education receiving facility and who visit hospitals.  Both seem to be out come of the responses.

The authors' discussions about KAP and raise in cases can not be made as 2nd time  data  not available.

The knowledge, attitude and Practices of the study sample can not be attributed the general population of Cameroon, Findings of the study sample , represent the behaviour a  better community with on line communication facility.

However, as per the findings there are 41/1006 respondents had ≥ 3 symptoms; Provided out of them  32(78.05%) of respondents who had more than 3 symptoms and did not call 1510,  this research may help to estimate how many more cases with symptoms  and not  positive to report in a  complex of several communities  of  Cameroon,  where there are    confirmed cases 8681, 4836 recovered and 212 deaths.

**Response**

Thank you the hypothetical estimate of suspected cases in yaounde and douala have been removed. Line 351-352

Reviewer #3: The authors made a compelling case for why we should consider their data as important information to have wide-spread testing in Cameroon. They also claimed that the data suggest a few improvements that could be made, especially in the gender-related correction of misperceptions about the COVID. I agree with them that this is relevant to public health.

However, it would have been interesting to discuss a bit about how we can apply the knowledge acquired in Cameroon for other African countries.

**Response**

The online system to identify the number of people who can have the symptoms and not go to the hospital. This is cheaper and more feasible to do in pandemics like this one.

Suggestion: improve the conclusion with recommendations, first, of how scholars could use the same research design to collect data in similar situations; and second, how learning what is happening in Cameroon helps us to understand the COVID experiences of comparable countries.

**Response**

This has been included in the manuscript

--------------------

Reviewer #1: The paper needs copy-editing to ensure the authors’ intentions are being clearly communicated.  Some comments follow:

Methodology

Line 22 – not clear “interacted face-to-face

**Response**

This applies to those rendering services to customers directly i.e customer services cashiers, hairdressers, traders, etc. Person to person interaction. Line 199

Line 24 – positive needs to be defined as well as good

**Response**

Thank you judgemental adjectives have been removed and replaced with high and low scores

Conclusion:

Line 28 (1st sentence of paragraph) needs clarification

**Response**

Thank you. It has been rephrased Line 31

Line 29 – Information that would be welcome in Background

Summary:

Lines 46 – use of satisfactory and good

Line 49 – needs to be clarified

Introduction

Line 61 – delete “seems to be very contagious”

**Response**

Thank you. It has been corrected

Line 66 – the phrase “ Some of these measures…” is insufficient. If there are others beyond what is listed, the reader would want to know them. If this is the complete list, delete “Some of”

**Response**

Thank you it has been listed. Line 78-85

Line 72 – The sentence needs some wordsmithing – ex The infection rate and ths the resources needed to battle this disease can be expected to increase exponentially

Response

Thank you. It has been adjusted. Line 88-90

Lines 74-83 – I would consider deleting most of this discussion of treatments as much of it is in flux. The main point here is that without treatments available many may turn to “non-standard options”

Study Design

Line 110 – what other cities/what populations

Line 116 – restricted to literate respondents

**Response**

Cameroonian residents from some cities in all the 10 regions of the country shown in the table below. We surveyed cities found in all the 10 regions of Cameroon. The following are the cities in the various regions (included in the appendix):

| Regions | Cities | Number of respondents |
| --- | --- | --- |
| Adamawa region | Ngaoundéré | 7 |
| West region | Bafang, Bafoussam, Baham, Dschang | 21 |
| Northwest region | Bambili, Bambui, Bamenda, Kumbo, Ndop | 41 |
| East region | Bertoua | 5 |
| Southwest region | Buea, Kumba, Limbe, mutenguene, Tiko | 111 |
| Extreme North region | Maroua | 6 |
| Littoral region | Douala, Nkongsamba | 152 |
| North region | Garoua, Garoua boulai, Doume | 10 |
| South region | Kribi, Sangmelima, Ebolowa | 13 |
| Centre region | Yaounde, Monatélé, Soa, Okola | 640 |

Symptomatology

There needs to a clearer link between the KAP and reported symptoms. Otherwise it doesn’t really belong in this paper. Why are we interested in the symptoms.  Was there any effort to explore their knowledge in terms of what symptoms are associated with CVD-19.

**Response**

Yes. The period and source through which they got the information contributed to their knowledge of the symptoms, those presenting with symptoms did they seek help or resorted to non-standard options? Table 8 has been added to respond to this and the aforementioned explanation.

Discussion

Last line of the first paragraph: this seems very speculative while at the same time does not really explain why women may report riskier behaviors. If the authors want to pursue this line of thinking, a more in-depth look is warranted. If correct and more clearly justified, it does provide program managers with a primary target audience to for behavior change initiatives

The last paragraph explores more deeply the issues of signs and symptoms and implications than KAP but if possible should be analyzed in light of the KAP objectives stated in the beginning.

**Response**

Thank you it has been done. Table 8 and Line 294-297

Reviewer #2: (No Response)

Reviewer #3: (No Response)

--------------------

Reviewer #1: As noted elsewhere, the authors have taken a bold step to look at the current pandemic from a behavioral perspective understanding the import that knowledge, attitudes and practices will play in reducing the transmission rate and lowering mortality.  Prior to publishing, I think the paper requires further thinking as to how the findings can advance Cameroon’s efforts to limit the impact of the infection.  I believe the data is there but more than just presenting the data is needed. A more robust discussion of interventions is needed as defined by the results of the study. If symptomatology is to be included, it needs to be linked to the stated objective of the paper, i.e. assess the knowledge, attitudes and practices.  Anchoring the paper more firmly in the Cameroon context, worrying less about the global situation, will also be helpful to fully understand how to move forward.

**Response**:

Thank you. The conclusions have been revised.

Reviewer #2: •    The title may be slightly modified to convey the main features of the study-whether it is on health population or affected peoples.

The concepts used in the study viz.  Knowledge/perception, Attitudes, Practices, Symptomatology and  co-morbidities may briefly be defined and explained from the scope of the current study.

**Response:**

Having symptoms and also comorbidities is high risk and not seeking help at the hospital would be much riskier. Worst case scenario resorting to traditional concoctions with no standard dosage and auto-medications. Line 379-380

Table-1: show (N=1006)

•    In the profession- who all include others. housewives are  part of the study or not.

**Response:**

Yes housewives are part of the study

•    In working environment-instead of Face to face interaction with customers  their place of  work may be mentioned. Face to face interaction (Yes/No) may be given  below  if data is available.

**Response:**

This applies to those rendering services to customers directly i.e customer services cashiers, hairdressers, traders, etc. Person to person interaction some of them don’t have a precise location of work (hawkers)

•    Following variables may be added: Education and family income/economic status, Disease related data (healthy/with early signs/confirmed of Covid-19)

**Response:**

Thank you . We did not take into consideration the educational status and economic status also we were looking at the suspected cases only given that no standard tests were done to confirm healthy and affected cases.

Reviewer #3: In this paper, the authors study the Knowledge, Attitudes, and Practices regarding the COVID-19 using an online survey in Cameroon. The authors recruited, from April 20 to May 20, 1006 respondents, using social media and websites, and the recruitment page directed the respondents to an online survey comprised of 32 questions. They find that respondents have a consistent knowledge about the COVID pandemics and that their symptom check and comorbidity studies match the prevalence of COVID-19 in the Cameroonian population. They also find that women tend to be less informed about COVID, which is interesting, as, around the world, women seem to care more about the disease than men.

Although the paper has several merits, I suggest a few revisions that would improve the article if adequately carried out. In the methods section, I detail my suggestions, guiding what I think could be done to improve the piece.

**Response:**

Thank you this has been corrected.

--------------------
